# Rheumatoid arthritis-associated depression: focusing on the interactions between mitochondria and endoplasmic reticulum

**DOI:** 10.3389/fphar.2025.1549060

**Published:** 2025-10-07

**Authors:** Mingqin Shi, Xinyao Li, Haimei Zhou, Zhenmin Li, Yuanyuan Wei, Zihui Wang, Yuqing She, Xuelian Zou, Xiangdian Xiao, Jiashun Zeng, Dongdong Qin

**Affiliations:** ^1^ Key Laboratory of Traditional Chinese Medicine for Prevention and Treatment of Neuropsychiatric Diseases, Yunnan University of Chinese Medicine, Kunming, Yunnan, China; ^2^ First Clinical Medical College, Yunnan University of Chinese Medicine, Kunming, Yunnan, China; ^3^ School of Traditional Chinese Medicine, Changchun University of Chinese Medicine, Changchun, Jilin, China; ^4^ School of Traditional Chinese Medicine, Qujing University of Medicine and Health Sciences, Qujing, Yunnan, China; ^5^ Department of Rheumatology and Immunology, The Affiliated Hospital of Guizhou Medical College, Guiyang, Guizhou, China

**Keywords:** rheumatoid arthritis, depression, mitochondria, endoplasmic reticulum, mitochondria-associated endoplasmic reticulum membranes

## Abstract

The interplay between mitochondria and endoplasmic reticulum (ER) is essential for cellular viability. The structures known as mitochondria-associated endoplasmic reticulum membranes (MAM) provide complicated connections between these organelles, which house a variety of proteins, each serving distinct roles across different cellular environments. Growing evidence indicates that disruptions in mitochondrial-ER interactions are linked to immune and inflammatory responses. The concurrent presence of rheumatoid arthritis (RA), an immune-mediated inflammatory condition, and depression has been well-documented. Alterations in proteins that mediate mitochondrial-ER interactions and MAM functionality are increasingly correlated with immune and inflammatory pathways. This suggests that a comprehensive understanding of disease mechanisms can be enhanced by examining the alterations in their intercommunication rather than viewing the organelles in isolation. In this review, we explore the pathophysiological mechanisms underlying RA in conjunction with depression, the relationships among mitochondria, the endoplasmic reticulum, mitochondrial-ER interactions, and their association with RA-associated depression, and propose that targeting MAM could offer a novel therapeutic approach for managing RA-associated depression.

## 1 Introduction

The hallmarks of RA, an autoimmune illness, include symmetrical, persistent synovitis, cartilage impairment, and joint deterioration, with clinical manifestations predominantly including arthralgia, edema, and deformity (Figus et al., 2021). According to global RA statistics, 17.6 million people worldwide were afflicted in 2020, with an age-standardized global frequency of 208.8 cases per 100,000, representing a 14.1% rise since 1990 (Global, 2023). Over the past thirty years, despite a reduction in the severity of RA attributable to advancements in treatment modalities and comprehensive disease management, the annual prevalence of RA has continued to rise (Finckh et al., 2022). It is noteworthy that patients with RA often experience significant emotional fluctuations due to chronic pain, limited mobility, side effects of medications (such as glucocorticoids-induced mood alterations), and impaired social functioning (Huyser and Parker, 1999). Depression, categorized as an affective disorder, is one of the most prevalent comorbidities associated with RA, leading to a deterioration in patients’ health status and profoundly affecting their quality of life (Ionescu et al., 2022; Liu et al., 2022). Depression is two to three times more common in RA patients than in the general population, according to research. Cross-sectional research carried out in 17 different countries have shown that the incidence of depression in RA patients ranges from 14% to 48% (Fakra and Marotte, 2021). In China, this figure escalates to as high as 48% (Fu et al., 2017). This high comorbidity rate suggests a complex bidirectional relationship between RA and depression. On one hand, the chronic inflammation and immune dysregulation associated with RA can directly drive central nervous system inflammation and neurotransmitter disturbances. On the other hand, depression can exacerbate the immune abnormalities of RA by activating neuroendocrine stress pathways, such as dysregulation of the hypothalamic-pituitary-adrenal (HPA) axis. This underscores the importance of addressing depression as a critical component in the treatment of RA (Uda et al., 2021).

In recent years, the interactions between organelles have attracted considerable attention, particularly concerning how the ER and mitochondria are related (Wu et al., 2023). Mitochondria-Endoplasmic Reticulum Contact Sites (MERCs) were first identified in electron microscopy images in the early 1950s (Bernhard et al., 1952). However, this structure was not identified as mitochondria-associated endoplasmic reticulum membranes (MAM) until the early 1990s (Simmen and Herrera-Cruz, 2018). Closely spaced mitochondria and ER make up the specialized membrane area known as MAM (10–80 nm apart) (Loncke et al., 2021). Studies have revealed that MAM is essential for controlling cellular homeostasis and function, including oxidative stress, lipid homeostasis, autophagy, and inflammatory responses (Chen et al., 2024; Missiroli et al., 2023; Rieusset, 2018; Missiroli et al., 2018; Lv et al., 2022). Given that the mechanisms regulated by MAM are intricately linked to RA and depression, investigating and establishing therapies that target MAM may represent a novel therapeutic strategy for managing RA-associated depression. The objective of this review is to clarify the pathophysiological mechanisms underlying RA-associated depression, the physiological characteristics of mitochondria and the ER, and the interactions among these organelles, with a focus on potential targeted therapeutic strategies for RA-associated depression.

## 2 Pathophysiological mechanism of RA-associated depression

The pathophysiological mechanisms underlying RA-associated depression remain inadequately elucidated, with inflammatory responses, oxidative stress, and autophagy identified as potential contributors. One characteristic of RA is the dysregulated expression of pro-inflammatory and anti-inflammatory mediators, which arises from an imbalance in immunological tolerance. Similarly, abnormal immune system and inflammatory pathway activation is linked to depression (Beurel et al., 2020). A key factor in the pathophysiology of RA is oxidative stress (da Fonseca et al., 2019), and research indicates that the inhibition of oxidative activation in RA may mitigate the onset of RA-associated depression (Omorog et al., 2018). Furthermore, autophagy is essential for the development, survival, and growth of different immune and non-immune cells, all of which are critical for the pathophysiology of RA (Karami et al., 2020). The dysregulation of these factors bears significant implications for the physiology and pathology of numerous neurological disorders, including depression.

### 2.1 Inflammatory responses

RA is among the most prevalent chronic autoimmune inflammatory disorders (Nerurkar et al., 2019). Investigations reveal that the dysregulation of both innate and adaptive immune responses is a critical determinant in this condition (Radu and Bungau, 2021). The immune system and neurons engage in bidirectional interactions, playing essential roles in maintaining organ homeostasis, orchestrating immune responses, and regulating inflammation. Immune cells can transmit stimuli to the nervous system, while signals from the central nervous system (CNS) influence immune cells via the peripheral nervous system (PNS) to modulate immune responses (Kabata and Artis, 2019; Bonaz et al., 2016). Depression is a multifactorial condition, and research has demonstrated that immune-mediated inflammation exerts both direct and indirect effects on cerebral function. The peripheral immune system communicates with the brain through neural and humoral pathways, including interactions across the blood-brain barrier and signaling from meningeal structures. Inflammation can impact the brain by altering monoamine neurotransmitter levels, neurotrophic factors, and synaptic plasticity (Brock et al., 2023). Recent studies have reported a notable increase in microglial density in brain regions lacking a blood-brain barrier, which remain persistently activated during chronic autoimmune arthritis. This observation suggests that sustained inflammation in RA may influence microglial activity in brain areas devoid of a blood-brain barrier, potentially leading to CNS-mediated manifestations such as depression (Matsushita et al., 2021).

RA, recognized as an immune-mediated inflammatory disorder, is marked by the upregulation of pro-inflammatory cytokines, including interleukin-1β (IL-1β), tumor necrosis factor-α (TNF-α), and IL-6 (Szekanecz et al., 2010; Firestein and McInnes, 2017). These cytokines are implicated in neuroinflammation within the brains of individuals suffering from depression, contributing to psychoneuroimmunological dysregulation (Nerurkar et al., 2019; Choy and Calabrese, 2018; Margaretten et al., 2011; Köhler et al., 2017). While the pathophysiology of depression is not fully elucidated, the interplay between immune-mediated inflammation and the underlying mechanisms of depression has garnered significant research interest. Numerous meta-analyses have revealed that individuals with depression exhibit heightened levels of pro-inflammatory markers (such as IL-6, IL-1β, TNF-α) in their peripheral circulation compared to healthy controls (Goldsmith et al., 2016; Köhler et al., 2017; Howren et al., 2009; Haapakoski et al., 2015). The parallels in inflammatory biomarker concentrations between depression and RA suggest a potential link between the inflammatory response and the onset of depression in the context of RA. Moreover, inflammatory mediators may adversely affect neurogenesis and neuroplasticity by diminishing neurotrophic factor levels in the brain (Calabrese et al., 2014). The hypothalamic-pituitary-adrenal (HPA) axis is intricately connected to depression, with studies indicating that chronic inflammatory stimuli in RA patients can elevate the secretion of corticotropin-releasing hormone (CRH), leading to increased cortisol production (Nerurkar et al., 2019). Sustained cortisol elevation can negatively impact brain function, precipitating depressive symptoms, particularly in RA patients with prolonged uncontrolled disease.

### 2.2 Oxidative stress

Oxidative stress represents a pathological condition characterized by a redox imbalance resulting from either an overproduction of reactive oxygen species (ROS) or a diminished antioxidant capacity. The free radicals produced during this process function as oxidants and inflammatory mediator integral to the pathology of RA (Salim, 2017; Bala et al., 2017). Inflammatory stimuli can activate the NADPH oxidase 2 (NOX2) complex, which serves as a primary source of ROS in specific cell types (Mittal et al., 2014). Research has established that oxidative stress is pivotal in the onset and exacerbation of chronic conditions such as RA (Ferreira et al., 2021). Investigations by Ge Zhang et al. (2012) have demonstrated oxidative damage in RA patients, while individuals with depression show compromised antioxidant defenses, indicating that oxidative stress may facilitate the emergence of depressive disorders. Furthermore, studies by Osarume Omorogbe et al. (2018) have illustrated that mitigating inflammatory responses in murine models through the inhibition of oxidative stress and the release of inflammatory cytokines can alleviate depressive-like behaviors. This leads to the hypothesis that targeting the oxidative stress pathways associated with RA may offer a viable strategy to attenuate the progression of RA-associated depression or alleviate depressive symptoms.

### 2.3 Autophagy

Autophagy serves as a cellular degradation mechanism that enables the recycling of cytoplasmic components to generate energy, thereby modulating immune responses to autoantigen through its influence on the development, survival, and proliferation of lymphocytes (Ohsumi, 2014). Disruption of autophagy can result in cellular dysregulation and disease progression. Research has established a significant association between autophagy and rheumatoid arthritis (RA). Specifically, the inhibition of persistently activated autophagy has been shown to diminish synovial inflammation and osteoclast in RA murine models, as well as to prevent structural damage (Lin et al., 2013). A study conducted by Jianting Wen et al. (2020) revealed that the expression of apoptosis and autophagy-related long non-coding RNAs (lncRNAs) in peripheral blood mononuclear cells from RA patients is markedly dysregulated, indicating the pivotal role of autophagy in RA pathophysiology. Moreover, a growing body of evidence from both clinical and preclinical investigations underscores the importance of autophagy regulation in the context of depression (Gassen and Rein, 2019). Mitochondrial autophagy facilitates the removal of dysfunctional mitochondria, thereby curtailing the production of ROS, inflammasome activation, and excessive neuroinflammation (Charmpilas et al., 2023). The accumulation of dysfunctional mitochondria within astrocytes exacerbates neuroinflammation, which subsequently contributes to the onset of depressive symptoms, while the activation of mitochondrial autophagy has been shown to mitigate mitochondrial damage in astrocytes (Yang et al., 2023). This suggests that mitochondrial autophagy may play a protective role against mitochondrial injury, thereby alleviating depressive symptoms. Under physiological conditions, autophagy can inhibit the excessive activation of the NLRP3 inflammasome and the secretion of pro-inflammatory cytokines. Autophagy is integral to the anti-inflammatory response by activating immune cells to produce pro-inflammatory mediators (Zhu and Liu, 2022). Importantly, autophagy is intricately linked to the activation of the NLRP3 inflammasome. Dysfunctional lysosomes within the autophagy-lysosome pathway hinder the degradation of the NLRP3 inflammasome, resulting in the production of pro-inflammatory factors, a process that can induce depressive-like behavior in murine models (Li et al., 2022).

## 3 The role of mitochondria and ER in RA-associated depression

### 3.1 Mitochondria in RA-associated depression

Mitochondria are semi-autonomous organelles distinguished by their double-membrane architecture, consisting of a matrix, an intermembrane gap, an inner membrane, and an outer membrane. The primary locations for energy production in the cellular environment are the mitochondria, which are the “powerhouses” of eukaryotic cells (Glancy, 2020). Through oxidative phosphorylation, the energy obtained from the oxidation and catabolism of nutrients, such as glucose and fatty acids, is transformed into adenosine triphosphate (ATP), thereby supplying energy for diverse cellular functions (Nunnari and Suomalainen, 2012). Moreover, mitochondria have a role in a wide range of biological functions, including signal transduction, apoptosis, redox reactions, and cell cycle regulation. The relative stability of mitochondrial number, shape, and spatial arrangement across different organs is known as mitochondrial homeostasis, and it is maintained by biological processes like biogenesis, fission, fusion, and autophagy order to meet the organism’s overall energy metabolism needs. The development and course of RA-associated depression are intimately related to the three features of mitochondrial biogenesis, alterations in mitochondrial dynamics, and mitochondrial autophagy.

Mitochondrial biogenesis denotes the process of mitochondrial formation, primarily involving the interaction of nuclear DNA (nDNA) and mitochondrial DNA (mtDNA). This relationship promotes the stability of the intracellular milieu by directing the self-renewal of organelles and enhancing the quality of mitochondria within the cell. Chronic inflammatory diseases like depression, RA, and pulmonary fibrosis can develop as result of nDNA degradation and mtDNA damage when mitochondrial biogenesis is impaired (Popov, 2020; Dang et al., 2020; Ogata et al., 2023).

Mitochondrial dynamics exemplify a state of dynamic equilibrium, characterized by the fission and fusion reactions that mitochondria go through to sustain their optimal quantity, morphology, and functionality. Investigations led by Shweta Khanna and colleagues have uncovered modifications in mitochondrial functional dynamics among patients with RA, indicating suggesting mitochondrial dysfunction could have a role in the functional differences seen in RA (Khanna et al., 2020). While excessive fission might hinder mitochondrial elongation and result in extended or stopped cell cycles, it is necessary for the quick clearance of damaged mitochondria. Dynamin 1-like protein (DNM1L), also known as dynamin-related protein 1 (Drp1), is an essential regulatory molecule for the preservation of regular mitochondrial function and plays a crucial role in mitochondrial fission (Wang et al., 2020). Following mitochondrial division, Drp1 migrates to the outer mitochondrial membrane, where it interacts with mitochondrial fission protein 1 (FIS1) to stimulate further mitochondrial division. Consequently, one possible treatment target for RA is the inhibition of Drp1-mediated mitochondrial fission (Yu et al., 2024).

One particularly kind of autophagy called mitochondrial autophagy breaks down and eliminates damaged mitochondria from the cytoplasm, preserving cellular homeostasis as well as the structural and functional integrity of the mitochondria. The PTEN-induced putative kinase 1 (PINK1) and PARK2 pathway has been extensively investigated in relation to mitochondrial autophagy (Truban et al., 2017). PINK1 is activated and localized at the outer mitochondrial membrane (OMM) in response to disruption of the mitochondrial transmembrane potential, which subsequently recruits and activates Parkin, further selectively enlisting autophagy receptors to trigger autophagy in the mitochondria (Lazarou et al., 2015). Research indicates that the levels of mitochondrial autophagy are significantly diminished in RA patients (Vasarmidi et al., 2018). In inflammatory settings, PINK1-mediated mitochondrial autophagy is suppressed (Willemsen et al., 2021). Downregulating Parkin expression can reduce the inflammatory response linked to arthritis by preventing p53 breakdown (Jung et al., 2017). Depression linked to RA is one of the more severe complications, with its pathogenesis associated with immune, inflammatory, and other factors (Smesam et al., 2022). Research has shown a connection between defects in mitochondrial autophagy and the activation of microglia, which can lead to neuroinflammation (Thangaraj et al., 2020; Agrawal and Jha, 2020). The absence of mitochondrial autophagy results in a rise in cytoplasmic reactive oxygen species (ROS) and damages of mtDNA, resulting in the inflammatory cytokine release (Harris et al., 2018).

### 3.2 The role of the ER in RA-associated depression

The ER serves as a multifunctional organelle integral to the synthesis and proper folding of the majorities of proteins within eukaryotic cells (Miglioranza Scavuzzi and Holoshitz, 2022; Martínez et al., 2018). Beyond this primary function, the ER is involved in many biological processes, including the preservation of intracellular calcium ions (Ca^2+^), lipid biosynthesis, protein transport, and the formation of autophagic vacuoles (Schwarz and Blower, 2016; Mo et al., 2024). Numerous elements, including inflammation, oxidative stress, dysregulation of calcium ions, and hypoxia can induce ER stress (ERS) by overwhelming the protein folding capabilities of the ER (Tabas and Ron, 2011). Prolonged and severe ERS can culminate in cellular apoptosis (Hetz et al., 2020). The hallmark of RA, an autoimmune disease, is immune cells’ overproduction of autoantibodies, which target normal tissues and instigate inflammatory cascades by releasing agents of inflammation, thereby exacerbating the progression of the disease. This pathological process is closely associated with ERS (Park et al., 2014). Research conducted by Liujun Wang et al. (2022) has demonstrated that ERS may activate autophagy via the IRE1/JNK signaling pathway and modulate the phenotypic transformation of fibroblasts within the synovial tissue of RA, underscoring the significant role of ERS in the pathophysiology of RA. In individuals suffering from depression, ERS and the unfolded protein response (UPR) are connected to the pathophysiological mechanisms underlying major depressive disorder (MDD) (Chen et al., 2023). The pathogenesis of MDD may involve a wide range of elements, such as oxidative stress, neuroinflammation, neuronal dysfunction, impaired neuroplasticity, and hormone abnormalities (Chang et al., 2024). Moreover, abnormal calcium signaling and ER-related stress responses have been linked to mood disorders, according to genetic and neuroimaging research (Allen et al., 2018; Ii Timberlake and Dwivedi, 2019). In conclusion, the intricate interaction between RA and depression may be significantly influenced by the ER.

### 3.3 Mitochondria-ER interactions

#### 3.3.1 MAM

MAM denotes a specialized membrane of the ER that is intricately linked to mitochondria, enabling interactions through a multitude of protein associations (Missiroli et al., 2018; Barazzuol et al., 2021). The structural heterogeneity of MAM signifies its extensive functional repertoire. At some locations, a communication nexus is established through the MERCs, which are the physical connections between the mitochondria and the smooth ER. The interactions involving ribosome-bearing ER and mitochondria are classified as ribosome-MERCs (Giacomello and Pellegrini, 2016). These contact sites are pivotal for lipid biosynthesis and transport, calcium ion uptake and release between the ER and mitochondria, signal transduction, and mitochondrial dynamics (Raby et al., 2024). Electron microscopy assessments reveal that the interspace between the ER and mitochondria measures approximately 10–30 nm (Katti et al., 2023). Morphologically, MAM can be classified based on the extent of coverage on the mitochondrial surface into three categories: isolated contact points, regions encompassing roughly 50% of the mitochondrial surface, and complete encasement (Jin et al., 2021). Important factors affecting the function of MAM in cellular processes include the number, length, and width of these contact zones (Wang et al., 2021a). MAM’s protein composition can change according to the normal and pathological conditions. This emphasizes how important it is to investigate how the MAM proteome changes under different circumstances and in different cell types in order to understand the complex processes that control MAM dynamics and functions (Wu et al., 2023).

#### 3.3.2 The structure of MAM

Membrane pieces from the ER and outer mitochondrial membrane that are connected by specific protein interactions make up MAM. The composition of MAM is notably intricate, incorporating several types of proteins that take part in numerous essential biological processes within the cell (Uoselis et al., 2023). Prominent MAM proteins include IP3R (Inositol 1,4,5-trisphosphate receptor), which serves as the primary mediator for calcium ion release from the endoplasmic reticulum. It facilitates efficient calcium ion transfer within the MAM region, thereby regulating critical processes of mitochondrial oxidative metabolism and energy production, which in turn affects lipid metabolism (Wang et al., 2021b). A channel protein on the outer mitochondrial membrane called the voltage-dependent anion channel (VDAC) is essential for controlling the flow of chemicals between the mitochondria and the cytoplasm (Zhang et al., 2024a). Glucose-regulated protein 75 (GRP75) is a molecule present in MAM that is crucial for proteins to fold correctly and the prevention of misfolded protein aggregation (Tiwary et al., 2021). Calreticulin (CALR), located within the ER lumen, is engaged in calcium ion binding and protein folding, and it interacts with GRP75 within the MAM (Jutzi et al., 2023). CAV1 (Caveolin-1), as a constituent of MAM, plays a significant regulatory role in cholesterol transport and membrane organization (Liu et al., 2024). The functional roles of MAM-related proteins are depicted in Table 1.

**TABLE 1:** The functional roles of MAM-related proteins.

Functional types	MAM-related proteins	Abbreviation	Biological functions	Ref.
Lipid metabolism	Acy1-Coenzyme A-cholesterol acyltransferase	ACAT	Synthesis of cholesteryl esters	Clapham (2007)
	Phosphatidylserine synthase 1 and 2	PSS1/2	Synthesis of phosphatidyl serine and phosphatidylcholine	Liang et al. (2022a)
	Cavsolin.1	CAV1	Regulation of cholesterol efflux	Willemsen et al. (2021)
Ca2+ homeostasis	Inositol1,4,5-trisphosphate receptor	IP3Rs	Regulation of calcium channels in ER	Ye et al. (2021)
	Voltage dependent anion channel 1	VDAC1	Regulation of calcium uptake channels	Liang et al. (2022b)
	Glucose-regulated protein 75	GRP75	Formation of VDAC1/GRP75/IP3R1 channel complex	Hayashi and Su (2007)
	Cyclophilin D	CYPD	Regulation of the MAM spatial structure	Hayashi (2019)
	Protein tyrosine phosphatase interacting protein 51	PTPIP51	Regulation of Ca2+ homeostasis	Hayashi (2015)
	ER resident protein 44	ERp44	Inhibition of lP3R	Mandelli et al. (2017)
	ER oxireductin1α	Ero1α	Maintainement of ER redox homeostasis	Hashimoto (2015)
	Sarco/ER Ca2+ ATPase	SERCA2b	Regulation of Ca2+ transportation into ER	Shimizu et al. (2013)
	FUN14 domain-containing protein 1	FUNDC1	Increases of mitochondrial Ca2+ content	Sabino et al. (2009)
	Mitofusin-2	MFN2	Regulation of mitochondrial fusion	Brimson et al. (2021)
Mitochondrial dynamics	Dynamin-related protein 1	Drp1	Regulation of mitochondrial fission	Antonini et al. (2009)
	Mitofusin-2	MFN2	Regulation of mitochondrial fusion	Ruscher et al. (2011)
	Inverted formin-2	INF2	Faciliation of initial mitochondrial	Geng et al. (2024)
	FUN14 domain-containing protein 1	FUNDC1	Regulation of mitochondrial fusion and fission	Antonini et al. (2009)
	Mitochondrial calcium uniporter	MCU	Decreases of mitochondrial division	Keller et al. (2023)
Inflammation	NOD like receptor (NLR) protein 3	NLRP3	Formation of the NLRp3 inflammasomes and MAMS	Cadwell (2016)
	Apoptosis-associated speck-like protein containing a CARD	ASC	Connection of NLRP3 and initiation of inflammatory signals	Missiroli et al. (2018)
Hypoxia	FUN14 domain-containing protein 1	FUNDC1	Induction of mitochondrial autophagy	Tao et al. (2024)
	Glucose-regulated protein 75	GRP75	Induction of cardiomyocyte apoptosis	Kato et al. (2014)

#### 3.3.3 The function of MAM in biological systems

MAM are integral to numerous biological functions, acting as pivotal sites for lipid biosynthesis and transport. They facilitate the exchange of lipids between the ER and mitochondria, encompassing the synthesis and translocation of phospholipids and cholesterol (Fernandes et al., 2023). By modulating intracellular calcium ion levels through inositol trisphosphate receptors (IP3R) and various calcium transport proteins, MAM is vital for calcium homeostasis, cellular signaling, and energy metabolism (Patergnani et al., 2011). As junctions between mitochondria and the ER, MAM may affect the generation and regulation of ROS, thus enhancing ROS production (Sarkar et al., 2014). Furthermore, MAM participates in the regulation of autophagy, supplying membranes and lipid substrates necessary for the formation of autophagosomes, which influences their growth and maturation (Xian and Liou, 2021). In the realm of inflammatory responses, an essential locus for the NLRP3 inflammasome’s assembly is MAM, which is intricately linked to the modulation of inflammatory processes (Missiroli et al., 2018). Numerous clinical illnesses, such as neurodegenerative diseases, inflammatory disorders, and metabolic syndromes, are linked to structural and functional anomalies in MAM. Consequently, MAM is essential for sustaining intracellular environmental equilibrium and influencing cellular outcomes.

#### 3.3.4 MAM in the context of RA-associated depression

Dysregulation of the ERS, mitochondrial dysfunction, and innate immune system abnormalities are closely intertwined with the pathophysiology of RA-associated depression. As a communication channel connecting the mitochondria and ER, alterations in the structure and functionality of MAM may be implicated in the etiology of RA-associated depression (Resende et al., 2020). Given that the specific role of MAM with relation to the RA-associated depression remains largely unexplored, it is imperative to clarify its influence on these psychopathological conditions.

The immune abnormalities associated with RA directly exacerbate neuroinflammation and neurotransmitter dysregulation in depression. Thus, neuroinflammation and immune dysregulation are critical mechanisms underlying depression in the context of RA. The MAM plays a pivotal role in modulating neuroinflammation and immune dysregulation. Inflammation is acknowledged as a vital component in the pathophysiology of RA in conjunction with depressive symptoms. Extended metabolic stress within the ER and mitochondria can initiate inflammatory cascades (Resende et al., 2020). Under conditions of chronic ER stress, it has been observed that the NF-kappaB-mediated anti-inflammatory signaling pathway becomes suppressed (Thoudam et al., 2016). It has been demonstrated that this inflammatory reaction affects the activation of the NOD-, LRR- and pyrin domain-containing 3 (NLRP3) inflammasome (Bronner et al., 2015). The NLRP3 inflammasome is a complex protein that stimulates Caspase-1 maturation. It is made up of the NLRP3 receptor on the ER side and the CARD-containing adaptor protein, often referred to as apoptosis-associated speck-like protein, on the mitochondrial side. Recent findings indicate that NLRP3 is repositioned from the ER to the MAM and promotes the inflammatory response by being activated by effectors produced from MAM (Liu et al., 2018). MAM is a crucial platform for the activation of the NLRP3 inflammasome and the subsequent release of mediators that promote inflammation (Thoudam et al., 2016). Changes at the ER-mitochondria interface may therefore be crucial to the onset and progression of several diseases, such as obesity, RA, and neurodegenerative disorders (Filadi et al., 2017).

## 4 Targeting MAM—a novel approach for managing RA-associated depression

**FIGURE 1:**
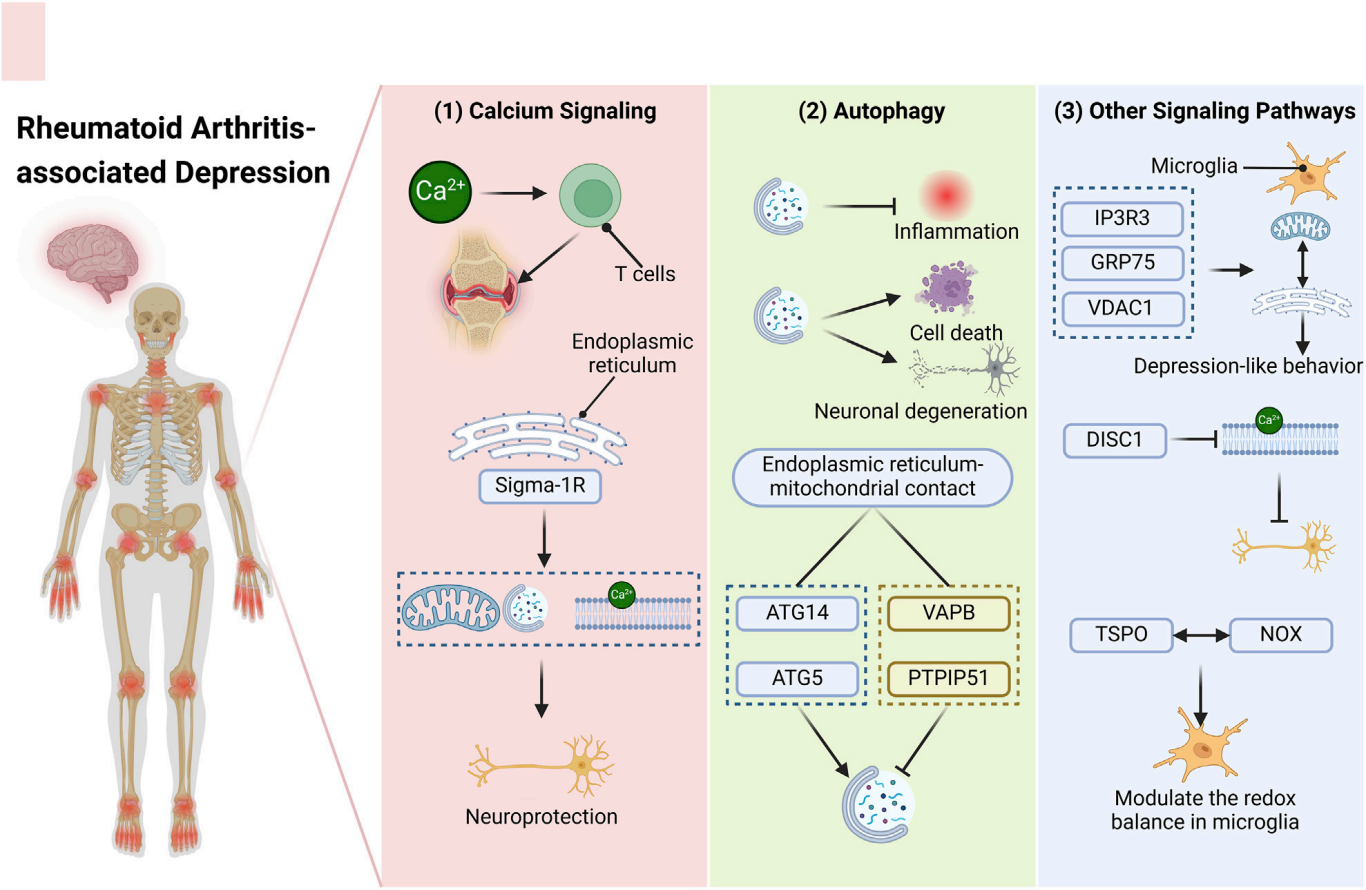
Targeting the MAM—An Innovative Approach for Addressing RA-associated Depression. The diverse signaling pathways present within the MAM may represent novel therapeutic targets for the management of RA-associated depression. (1) Calcium ions play a crucial role in modulating T cell activity and mediating synovial inflammation in RA patients during inflammatory processes. A calcium-sensitive chaperone situated in the ER membrane, Sigma-1R, can initiate mitochondrial autophagy and regulate calcium transport, thereby providing neuroprotective benefits. (2) Autophagy exhibits a dual function in the context of RA and depression. It can mitigate inflammation on one hand, while potentially inducing cell death and neuronal degeneration on the other hand. The effects of various stimuli can differ significantly. At the contact sites between the ER and mitochondria, ATG14 and ATG5 facilitate the formation of autophagosomes, whereas VAPB and PTPIP51 act to inhibit this process. (3) Moreover, additional signaling pathways may also emerge as potential therapeutic targets. The tripartite complex comprising IP3R3, GRP75, and VDAC1 within the MAM can strengthen the interaction between the ER and mitochondria in microglial cells, contributing to mood disorders like depression-like behavior. DISC1, which is enriched in the MAM, disrupts calcium transfer, thereby impacting neuronal functionality. A MAM-resident protein, TSPO, interacts with NOX to modulate the redox balance in microglia. Abbreviations: RA, rheumatoid arthritis; MAM, mitochondria-associated endoplasmic reticulum membranes ER, endoplasmic reticulum; ATG14, autophagy related 14 Gene; ATG5, autophagy related 5 Gene; VAPB, vesicle-associated membrane protein-associated protein B/C; PTPIP51, protein tyrosine phosphatase interacting protein 51; IP3R3, inositol 1,4,5-trisphosphate receptor type 3 Gene; GRP75, 75-kDa glucose-regulated protein; VDAC1, voltage-dependent anion channel; DISC1, disrupted-in-schizophrenia-1; TSPO, translocator protein; NOX, reduced nicotinamide adenine dinucleotide phosphate oxidase.

The importance of ER-mitochondrial interaction sites in the development of neuroinflammation has been emphasized in recent research. However, the exploration of MAM as potential therapeutic targets for RA-associated depression remains inadequately addressed. This study aims to identify novel therapeutic targets for RA-associated depression by examining the modulation of pertinent signaling pathways within MAM (Figure 1).

### 4.1 Targeting calcium signaling in MAM

Calcium ions (Ca^2+^) are released from the ER and utilize MAM as pathways to transfer to the mitochondria (Rizzuto et al., 1998). As a vital second messenger in cellular signaling, a pronounced concentration gradient exists between intracellular and extracellular Ca^2+^ levels, necessitating significant energy expenditure by cells to sustain this gradient (Clapham, 2007). Ca^2+^ is integral to inflammation and inflammatory diseases (Liang et al., 2022a). It has been suggested that during inflammatory responses, Ca^2+^ mediates synovial inflammation in RA patients and controls T cell metabolism of arachidonic acid (Ye et al., 2021). Furthermore, extensive evidence indicates that Ca^2+^ facilitates the infiltration of various immune cells during RA progression, resulting in dysregulated inflammatory responses (Liang et al., 2022b). This underscores the central role of Ca^2+^ in inflammatory processes, particularly in RA.

Calcium channels mediate the release of intracellular Ca^2+^ or the input of extracellular Ca^2+^ and are widely expressed on cell membranes, ER membranes, and mitochondrial membranes. Consequently, Ca^2+^ channels are essential for all biological functions. The Sigma-1 receptor (Sigma-1R) is abundantly present at the ER-mitochondria interface and is instrumental in modulating organelle Ca^2+^ signaling and bioenergetics (Hayashi and Su, 2007). Furthermore, by facilitating the transmission of stress signals from the ER to the nucleus, Sigma-1R raises intracellular antioxidant protein levels (Hayashi, 2019). Sigma-1R is pivotal in the context of neuropsychiatric disorders (Hayashi, 2015). Research has indicated that Sigma-1R is implicated in mood disorders, potentially linked to genetic variations in SIGMAR1 and many antidepressants’ interactions with these receptors (Mandelli et al., 2017; Hashimoto, 2015). Investigations by Shimizu et al. have demonstrated that plasma levels of Sigma-1R rise in patients with MDD following antidepressant therapy (Shimizu et al., 2013). Additionally, Sigma-1R knockout mice display depressive-like phenotypes (Sabino et al., 2009). Several pharmacological agents with affinity for Sigma-1R, including donepezil, escitalopram, fluvoxamine, and fluoxetine, have exhibited antidepressant-like and neuroprotective properties (Hashimoto, 2015). Research conducted by James Michael Brimson et al. has demonstrated that sigma-affinity drugs may confer protective effects through interactions with the Sigma-1R, potentially by activating mitochondrial autophagy, alleviating ER stress, and regulating Ca^2+^ transportation (Brimson et al., 2021). Numerous Sigma-1R agonists exhibit neuroprotective properties under conditions of neuronal stress (Antonini et al., 2009; Ruscher et al., 2011). Therefore, Sigma-1R may represent a pivotal ligand for targeting calcium signaling within MAM in forthcoming therapeutic strategies.

### 4.2 Targeting autophagy in MAM

Autophagy represents a fundamental molecular pathway essential for the preservation of cellular and physiological equilibrium (Geng et al., 2024). It exhibits a bifunctional role in the context of RA and depression (Keller et al., 2023). On the one hand, autophagy lowers inflammation by regulating the release of inflammatory-promoting cytokines (Cadwell, 2016; Tao et al., 2024). Conversely, it may also exacerbate the progression of these diseases. In instances of severe ERS, the suppression of autophagy can lead to increased cell death in RA (Kato et al., 2014). In contrast, heightened neuronal autophagy may aggravate the advancement of depression by diminishing levels of brain-derived neurotrophic factor (BDNF) (Zhang et al., 2023). The interaction between mitochondria and the ER is pivotal in the regulation of both autophagy and mitophagy, with diverse stimuli eliciting distinct responses (Son et al., 2012). Contact areas between the ER and mitochondria are essential for the development of autophagosomes, according to research by Maho Hamasaki et al., and primarily linked to the markers ATG14 and the autophagosome formation marker ATG5 (Hamasaki et al., 2013). However, investigations by Patricia Gomez-Suaga et al. suggest that the lack of endoplasmic reticulum-mitochondria contacts can stimulate autophagy, predominantly associated with the depletion of VAPB or PTPIP51 (Gomez-Suaga et al., 2017). In conclusion, these studies look into the possibility of therapeutically targeting molecules that control autophagy at the MAM, underscoring their significant promise as therapeutic targets for treating RA-associated depression.

### 4.3 Targeting other signaling pathways in MAM

Beyond calcium signaling and autophagy, recent findings indicate that signaling pathways at the MAM can modulate immune responses and cellular senescence, thereby offering a novel therapeutic strategy aimed at MAM. A study has revealed that in the hippocampus of mice with depression, ER stress and mitochondrial damage occur, leading to a significant increase in mitochondrial-ER interactions and the enrichment of MAM proteins (Zhang et al., 2024b). The connections between the ER and mitochondria in microglia within the hippocampus can be enhanced by the formation of a tripartite complex consisting of IP3R3, GRP75, and VDAC1 within the MAM. This suggests that MAM may serve as a potential target for improving depression. Disrupted-in-schizophrenia 1 (DISC1) is essential for regulating the dynamics of mitochondria in axons and dendrites, which promotes communication between the ER and mitochondria. As a protein linked to mood disorders, DISC1 is notably concentrated in the MAM (Norkett et al., 2016). Investigations by Sung Jin Park et al. (2017) demonstrated that the impairment of DISC1 disrupts Ca^2+^ transfer, resulting in the accumulation of Ca^2+^ within mitochondria following oxidative stress, which subsequently impacts mitochondrial functionality in neurons. The 18 kDa translocator protein (TSPO), a resident protein of the MAM, is found in the outer mitochondrial membranes of the central and peripheral nerve systems. TSPO is frequently employed as a neuroinflammation biomarker in preclinical and clinical neuroimaging investigations. Studies conducted by Meredith K Loth et al. (2020) suggest that TSPO may interact with NADPH (nicotinamide adenine dinucleotide phosphate) oxidase, thereby modulating the redox homeostasis of microglia. The ER membrane protein PDZD8 plays a crucial role in the formation of contact sites between mitochondria and the endoplasmic reticulum. Research by Koki Nakamura et al. (2025) has revealed that PDZD8 forms a novel ER-mitochondrial anchoring complex with FKBP8 in mammalian cells, which regulates mitochondrial morphology. This finding offers new insights into the regulatory mechanisms between mitochondria and the endoplasmic reticulum and provides potential molecular targets for studying diseases associated with mitochondrial and endoplasmic reticulum dysfunction, such as RA-associated depression.

Nevertheless, current research primarily focuses on preclinical studies, with limited participation in clinical trials. More clinical trials should be designed in the future to explore the mechanisms and therapeutic effects of targeting MAM and their signaling pathways in RA-associated depression. This will help to facilitate the transition of relevant research from the laboratory to the bedside and provide novel strategies for treating RA-associated depression.

## 5 Conclusion and prospect

In recent decades, there has been an escalating interest in intracellular communication, particularly through the intricate interactions between organelles. The contact sites established between the ER and mitochondria—two organelles essential for sustaining intracellular homeostasis—are linked to various signaling crosstalk pathways. Current evidence suggests that the critical intracellular signaling platform formed by MAM experiences alterations during the progression of diverse immune and inflammatory diseases. Emerging studies increasingly highlight that the dynamics of organelles, autophagy, and metabolic signaling pathways associated with MAM present substantial potential for the identification of novel therapeutic targets for immune and inflammatory conditions. RA, particularly when accompanied by depression, represents a category of diseases intricately connected to immune and inflammatory processes, yet its treatment has been insufficiently investigated. Future pharmacological development aimed at MAM signaling pathways may offer innovative strategies for the management of this complex condition.
